# Our Eighty-Ninth Annual Meeting

**Published:** 1911-03

**Authors:** 


### Our Eighty-ninth Annual Meeting

"THERE were giants in the earth in those days." America is comparatively a young country, and her medical institutions are not of those first established, however, a medical society which celebrates its eighty-ninth annual meeting deserves from years alone careful consideration. The Medical Society of the County of Erie deserves thoughtful appreciation for the years, its work, and more than these, for its aspirations.

In making his annual address, retiring President Wende spoke with earnestness, wisdom and inspiration showing the sagacity of the society in its previous selection for its highest honors. proving that this high distinction had been fully appreciated, and that the appreciation had found expression in unusually intelligent and successful promotion of the best interests of the society. The reports of the various officers and committees furnish the evidence in detail of the success of this remarkable growth; in new members—in power; in unity of purpose—in effectiveness; in record of work accomplished—in persistency; in ambitious plans for future labors, plans involving important questions, hygienic, economic, social—whereby may come inspiration for many years.

In our judgment the high note of President Wende's address was given in his keen analysis of the obstacles to the altruistic work of the medical profession, and his wise suggestions as to how those obstacles are to be met and overcome. In short, medical societies must have more public confidence and support in order that better work may be done, and must deserve more public confidence before they have the right to expect the same. By raising the standard of medical preparedness, competency, efficiency; by excluding, discouraging, eliminating the commercialised or commercialising members of our profession; by convincing those in authority that the only successful preparation and foundation for medical education or medical career is a moral character of the highest ideals—this is the way to win public confidence, to achieve greater success—is the dictum of president Wende.

We believe completely that here is a case in which thorough insight gives wise foresight, and we heartily congratulate all concerned on the record of the past, on the immediate state, and on the future prospects. "The tree shall be known by its fruits."

